# The *sbiTRS* Operon Contributes to Stenobactin-Mediated Iron Utilization in Stenotrophomonas maltophilia

**DOI:** 10.1128/spectrum.02673-22

**Published:** 2022-12-01

**Authors:** Cheng-Mu Wu, Li-Hua Li, Yen-Ling Lin, Chao-Jung Wu, Yi-Tsung Lin, Tsuey-Ching Yang

**Affiliations:** a Department of Biotechnology and Laboratory Science in Medicine, National Yang Ming Chiao Tung University, Taipei, Taiwan; b Department of Pathology and Laboratory Medicine, Taipei Veterans General Hospital, Taipei, Taiwan; c School of Medical Laboratory Science and Biotechnology, College of Medical Science and Technology, Taipei Medical University, Taipei, Taiwan; d Division of Infectious Diseases, Department of Medicine, Taipei Veterans General Hospital, Taipei, Taiwan; e Department of Medicine, National Yang Ming Chiao Tung University, Taipei, Taiwan; University of Maryland School of Pharmacy

**Keywords:** *Stenotrophomonas maltophilia*, stenobactin, two-component regulatory system, iron homeostasis, ABC-type efflux pump

## Abstract

Iron is an essential micronutrient for various bacterial cellular processes. Fur is a global transcriptional regulator participating in iron homeostasis. Stenotrophomonas maltophilia is a ubiquitous environmental bacterium that has emerged as an opportunistic pathogen. To elucidate the novel regulatory mechanism behind iron homeostasis in S. maltophilia, wild-type KJ and KJΔFur, a *fur* mutant, were subjected to transcriptome assay. A five-gene cluster, *sbiBA-sbiTRS*, was significantly upregulated in KJΔFur. SbiAB is an ATP type efflux pump, SbiT is an inner membrane protein, and SbiSR is a two-component regulatory system (TCS). The *sbiTRS* operon organization was verified by reverse transcription-PCR (RT-PCR). Localization prediction and bacterial two-hybrid studies revealed that SbiT resided in the inner membrane and had an intramembrane interaction with SbiS. In iron-replete conditions, SbiT interacted with SbiS and maintained SbiSR TCS in a resting state. In response to iron depletion stress, SbiT no longer interacted with SbiS, leading to SbiSR TCS activation. The iron source utilization assay demonstrated the contribution of SbiSR TCS to stenobactin-mediated ferric iron utilization but notto the utilization of hemin and ferric citrate. Furthermore, SmeDEF and SbiAB pumps, known stenobactin secretion outlets, were members of the SbiSR regulon. Collectively, in an iron-depleted condition, SbiSR activation is regulated by Fur at the transcriptional level and by SbiT at the posttranslational level. Activated SbiSR contributes to stenobactin-mediated ferric iron utilization by upregulating the *smeDEF* and *sbiAB* operons. SbiSR is the first TCS found to be involved in iron homeostasis in S. maltophilia.

**IMPORTANCE** Therapeutic options for Stenotrophomonas maltophilia infections are limited because S. maltophilia is intrinsically resistant to several antibiotics. Iron is an essential element for viability, but iron overload is a lethal threat to bacteria. Therefore, disruption of iron homeostasis can be an alternative strategy to cope with S. maltophilia infection. The intricate regulatory networks involved in iron hemostasis have been reported in various pathogens; however, little is known about S. maltophilia. Herein, a novel *sbiTRS* operon, a member of Fur regulon, was characterized. SbiT, an inner membrane protein, negatively modulated the SbiSR two-component regulatory system by intramembrane protein-protein interaction with SbiS. In response to iron-depleted stress, SbiSR was activated via the regulation of Fur and SbiT. Activated SbiSR upregulated *smeDEF* and *sbiAB*, which contributed to stenobactin-mediated ferric iron utilization. A novel *fur-sbiT-sbiSR-smeDEF/sbiAB* regulatory circuit in S. maltophilia was revealed.

## INTRODUCTION

Iron is an essential micronutrient in nearly all living organisms. Iron mainly exists in two forms, namely, ferric iron (Fe^3+^) and ferrous iron (Fe^2+^). Ferric iron has a low solubility under biological pH and aerobic conditions (1). Ferrous iron performs vital biological functions at low levels but can cause toxicity at higher cellular concentrations. Excess ferrous iron reacts with hydrogen peroxide through Fenton reaction to form hydroxyl radicals, which can damage virtually all types of macromolecules, such as carbohydrates, nucleic acids, lipids, and amino acids (2). Consequently, bacteria have evolved several mechanisms of intracellular iron homeostasis. To deal with excess intracellular ferrous iron, ferrous iron export and storage systems work to maintain the intracellular iron levels at homeostatic concentrations (3). However, bacteria also harbor several iron acquisition systems to overcome the shortage of intracellular iron. There are two main types of iron acquisition systems in Gram-negative bacteria. First, in response to iron-depleted stress, bacteria secrete iron chelators (including siderophores, hemophores, and citrate) to scavenge ferric iron or hemin from the extracellular environment. Second, bacteria can directly capture iron-containing molecules (such as hemoglobin, transferrin, and lactoferrin) from the environment via specific TonB-dependent receptors. In addition, bacterial iron storage proteins (such as ferritin and bacterioferritin) provide intracellular iron reserves for use when external supply is restricted (4). These iron homeostasis-associated systems must be precisely controlled to maintain intracellular free iron at a functional but not toxic level (5).

Fur (ferric uptake regulator) is a highly conserved master regulator of iron homeostasis in many bacteria (6). It is a dimeric metal-dependent DNA-binding protein, utilizing Fe^2+^ as a cofactor to repress transcription of the iron homeostasis-associated genes (7). When the intracellular Fe^2+^ level is sufficient, the active Fur-Fe^2+^ complex binds to a Fur box, which is located in the vicinity of the promoter region of Fur-regulated genes, thereby repressing the expression of these genes. Once the intracellular Fe^2+^ level decreases, the iron-free Fur protein loses its affinity to the Fur box, derepressing the expression of Fur-regulated genes. Thus, cells lacking Fur experience iron overload and are, therefore, prone to oxidative damage and mutagenesis (8). In addition, other regulatory components involved in the regulation of iron homeostasis have also been identified, including transcriptional regulators (6), sigma factors (9), small RNAs (10), and two-component regulatory systems (11).

The two-component regulatory system (TCS) forms an intricate signal network to cope with different environmental stresses (12). A canonical TCS consists of a membrane-bound sensor kinase (SK) and cytoplasmic response regulator (RR). In general, genes encoding SK and RR are organized into an operon. In response to environmental stimuli, SK triggers a signaling cascade that leads to phosphorylation of RR. Phosphorylated RR can transcriptionally regulate gene expression as well as modulate protein function through protein-protein interactions (13). TCSs participate in the regulation of nutrient acquisition, virulence, antibiotic resistance, response to various stresses, and numerous other pathways in diverse bacteria (12). TCS has been reported to be involved in iron homeostasis, including HrrS/HrrA and ChrS/ChrA in Corynebacterium diphtheriae (14), VirR/VirS in Clostridium perfringens (15), and EnvZ/OmpR in Escherichia coli (16).

Stenotrophomonas maltophilia, an aerobic, nonfermentative, Gram-negative bacillus, inhabits multiple niches under disparate environmental conditions and is regarded as an opportunistic pathogen (17). The prevalence of nosocomial infections caused by S. maltophilia has increased remarkably among immunocompromised patients with indwelling medical devices and prolonged hospitalization. The treatment of S. maltophilia infection is particularly challenging because of its intrinsic and acquired resistance to several antibiotics, such as aminoglycosides, β-lactams, and macrolides (18). Iron homeostasis is crucial for pathogens; hence, a comprehensive understanding of iron homeostasis may help control these infections (19).

Like in the case of most pathogens, S. maltophilia has developed several mechanisms to maintain iron homeostasis. In response to iron depletion stress, S. maltophilia synthesizes the catechol-type siderophore stenobactin via the *entCEBB’FA* system (20). SmeYZ, SmeDEF, and SbiAB pumps are known outlets for stenobactin export into the extracellular environment (21). Once bound to ferric iron, ferri-stenobactin is taken up by the TonB-dependent outer membrane receptor FepA (22). Additional iron acquisition systems documented in S. maltophilia include the PacIRA system for the uptake of Pseudomonas aeruginosa pyrochelin (23), the FciTABC-FeoABI system for ferric citrate (24), and the HemP-HemA system for hemin (25).

Fur, AmpR, and HemP are three known regulators involved in the regulation of iron homeostasis in S. maltophilia. Fur is involved in iron homeostasis, biofilm formation, oxidative stress response, and virulence (26). AmpR is a well-known LysR-type transcriptional regulator responsible for the expression of L1 and L2 β-lactamases (27). Recently, we have revealed the role of AmpR in stenobactin synthesis (20). HemP, a transcriptional factor, negatively regulates hemin acquisition in S. maltophilia (25). In this study, we report a novel TCS, namely, SbiS/SbiR, and demonstrate its role in the iron homeostasis.

## RESULTS

### *sbiT-sbiR-sbiS* form an operon.

**FIG 1:**
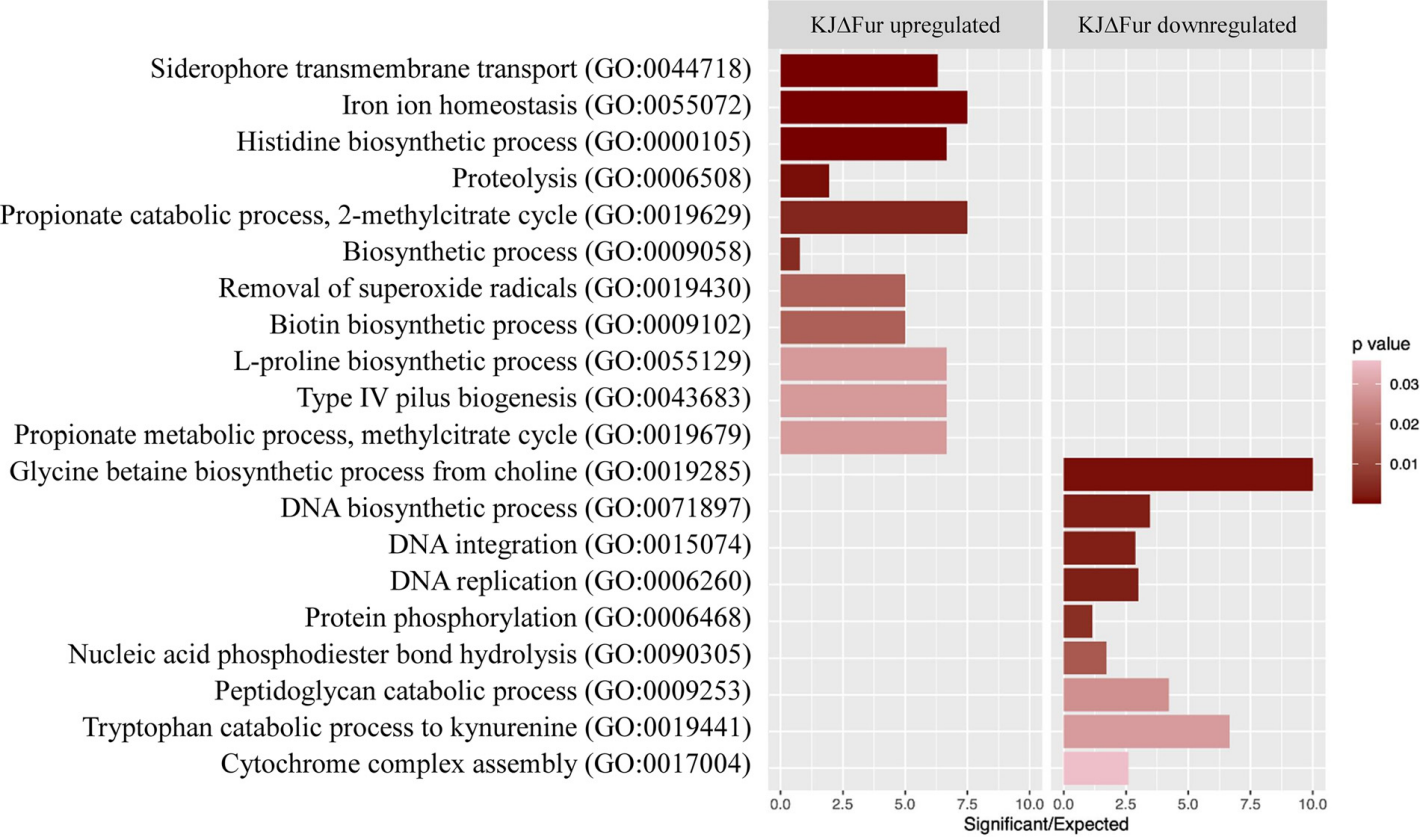
Gene ontology classification of KJ and KJΔFur transcriptomes. Gene enrichment analysis of the differentially expressed genes (DEGs) was carried out using topGO package with Fisher exact test and weighted 01 algorithm. The GO terms with *P* < 0.01 were selected as significantly enriched functional groups.

Ferric uptake regulator (Fur) is a global transcription factor that regulates iron homeostasis in bacteria (6). Not surprisingly, some regulatory components are under the regulation of Fur. With the aim of disclosing novel regulatory systems for iron homeostasis in S. maltophilia, we performed transcriptome sequencing (RNA-seq) transcriptome analyses of wild-type KJ and its *fur* isogenic mutant, KJΔFur, in the stationary phase of growth. We defined differential expression as a relative change in transcript level (transcripts per kilobase million) equal to or greater than 3-fold. Approximately 805 differentially expressed genes (DEGs) were found in KJΔFur cells, of which 414 were upregulated and 391 were downregulated (see Table S1 in the supplemental material). As expected, the genes involved in iron homeostasis were upregulated; however, the genes related to synthetic and catabolic processes were also upregulated. In contrast, the genes coding for DNA replication and integration were downregulated (Fig. 1).

**FIG 2:**
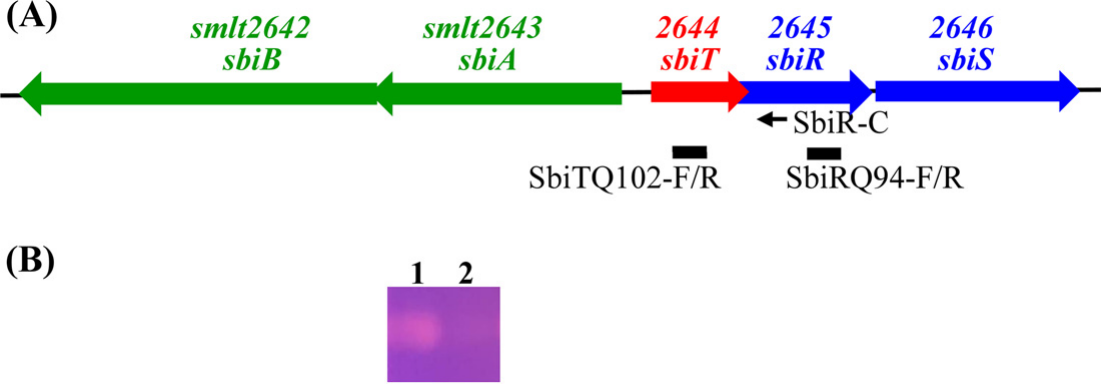
*SbiT*, *sbiR*, and *sbiS* form an operon. (A) The genetic organization of the *sbiB-sbiA-sbiT-sbiR-sbiS* genes cluster of S. maltophilia. Arrows represent open reading frames (ORFs) and the direction of transcription. Small black arrow indicates the position of primer SbiR-C used for reverse transcription. The solid lines represent the PCR amplicons amplified using the SbiTQ102-F/R and SbiRQ94-F/R primer sets. (B) Agarose gel electrophoresis of the PCR products. DNA-free RNA was purified from logarithmically-grown KJΔFur cells, and cDNAs were obtained by reverse transcription using the primer SbiR-C. The cDNA was used as template for PCR with the primers indicated. Lane 1, primers SbiTQ102-F and SbiTQ102-R; Lane 2, primers SbiRQ94-F/R. The SbiRQ94-F/R primer sets were used as a control for a check of DNA contamination during cDNA preparation.

Stenobactin is the sole known siderophore in S. maltophilia (20). Some genes involved in stenobactin-mediated ferric iron acquisition have been revealed, including stenobactin synthesis genes (*entCEBB’FA*) (20), stenobactin secretion genes (*smeYZ*, *smeDEF*, and *sbiAB*) (21), and ferri-stenobactin uptake gene (*fepA*) (22). These genes’ expression in KJ and KJΔFur revealed by transcriptome analysis was summarized in Table 1. Of them, the five-gene cluster *sbiB*-*sbiA*-2644-2645-2646 attracted our attention (Fig. 2A). The proteins encoded by these five genes included a two-component regulatory system (Smlt2645-Smlt2646), an inner membrane protein (Smlt2644), and an ABC-type SbiAB efflux pump, which acts as an outlet for stenobactin secretion (21). Given this genomic organization, we designated Smlt2644, Smlt2645, and Smlt2646 as *sbiT*, *sbiR*, and *sbiS*, respectively. Figure 2A shows the genomic architecture of the *sbiBATRS* locus. Since there was a 4-bp overlap between *sbiT* and *sbiR* genes, we speculated that *sbiT* and the *sbiRS* TCS form an operon. Reverse transcription-PCR (RT-PCR) was performed to verify the presence of the *sbiTRS* operon (Fig. 2B).

**TABLE 1:** The differentially expressed genes of KJ and KJ**Δ**Fur, selected from transcriptome analysis

Gene	TPM^*a*^		Fold change^*b*^	Encoded protein
	KJ	KJΔFur		
Snlt2642 (*sbiB*)	12.76	52.68	+4.12	SbiB, ABC-type transporter
Smlt2643 (*sbiA*)	11.19	41.58	+3.72	SbiA, membrane fusion protein
Smlt2644 (*sbiT*)	5.97	35.80	+5.99	SbiT, transmembrane protein
Smlt2645 (*sbiR*)	8.42	64.40	+7.65	SbiR, response regulator of TCS
Smlt2646 (*sbiS*)	12.96	79.10	+6.11	SbiS, sensor kinase of TCS
Smlt2817 (*entA*)	0.1	105.43	+1,054.29	EntA, 2,3-dihydro-2,3-dihydroxybenzoate dehydrogenase
Smlt2818 (*entF*)	3.45	396.46	+114.84	EntF, enterobactin synthase component F
Smlt2819 (*entB*’)	3.87	304.14	+78.68	EntB′, C terminus of enterobactin synthase component B
Smlt2820 (*entB*)	5.86	1,342.87	+229.22	EntB, N terminus of enterobactin synthase component B
Smlt2821 (*entE*)	22.65	1,049.43	+46.32	EntE, 2,3-dihydroxybenzoate-AMP ligase
Smlt2822 (*entC*)	1.34	286.13	+213.10	EntC, isochorismate synthase
Smlt2201 (*smeY*)	213.93	387.94	+1.81	SmeY, membrane fusion protein
Smlt2202 (*smeZ*)	148.87	235.66	+1.58	SmeZ, RND-type transporter
Smlt4070 (*smeF*)	25.10	101.70	+4.05	SmeF, outer membrane protein
Smlt4071 (*smeE*)	81.11	174.32	+2.15	SmeE, RND-type transporter
Smlt4072 (*smeD*)	53.38	198.59	+3.72	SmeD, membrane fusion protein
Smlt1426 (*fepA*)	26.64	3,767.91	+141.42	FepA, TonB-dependent OMP

a TPM, transcripts per kilobase million.

b Negative fold changes represent genes that were significantly downregulated in response to *fur* deletion, whereas positive fold changes represent upregulation in response to *fur* deletion.

**FIG 3:**
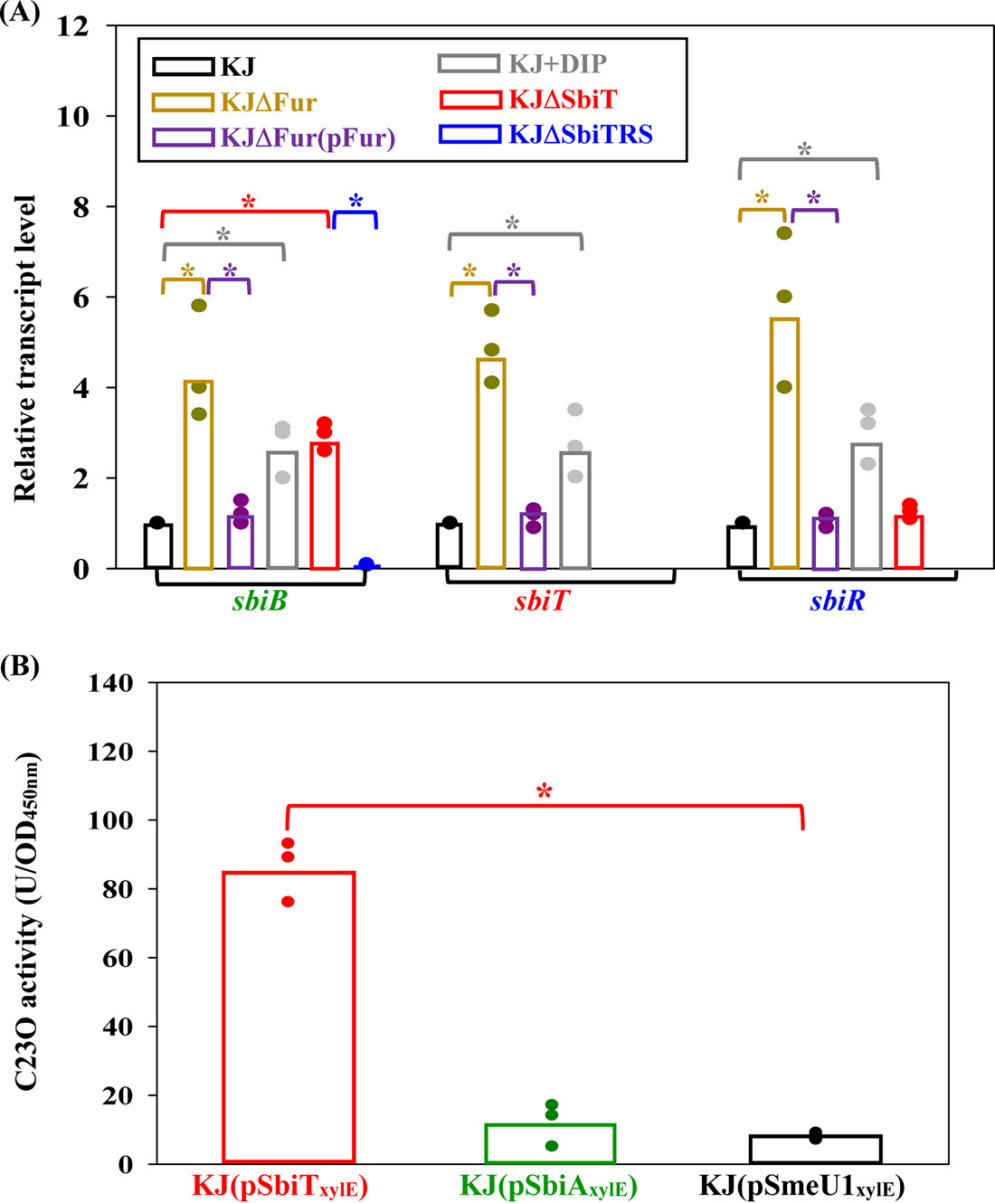
The expression of *sbiBA-sbiTRS* cluster. Data are represented as the means of values from three independent experiments. Bars represent the means of values from three independent experiments. Error bars represent the standard deviation for triplicates. *, *P *< 0.01; significance calculated by Student's *t* test. (A) Regulation of *sbiBA*-*sbiTRS* cluster expression. The overnight-cultured bacterial cells as indicated were inoculated into fresh LB broth at an initial OD_450_ of 0.15. The DIP was added to a final concentration of 30 μg/mL. After 15 h culture, DNA-free RNA was isolated. The levels of transcripts as indicated were determined by qRT-PCR. The primers used were listed in Table S3 in the supplemental material. Relative transcript levels were calculated using the 2^−ΔΔ^*^CT^* method with wild-type KJ transcript level set as 1. (B) The expression of *sbiTRS* and *sbiAB* operons in logarithmically grown KJ cells. The overnight-cultured bacterial cells as indicated were inoculated into fresh LB broth at an initial OD_450_ of 0.15. After 5 h culture, the C23O activities were determined.

The transcript levels of *sbiB*, *sbiT*, and *sbiR* in stationary-phase KJ and KJΔFur were determined using quantitative real-time PCR (qRT-PCR) to verify the results of the transcriptome assay (Fig. 3A). In addition, the impact of iron depletion on the expression of *sbiAB* and *sbiTRS* operons was investigated. The *sbiB*, *sbiT*, and *sbiR* transcripts showed 2.73 ± 0.46-, 2.73 ± 0.6-, and 2.9 ± 0.9-fold increases in levels, respectively, in the presence of 30 μg/mL 2,2′-dipyridyl (DIP), a ferrous iron chelator (Fig. 3A).

Next, the expression levels of the *sbiTRS* and *sbiAB* operons in logarithmically grown KJ cells were determined. The promoter-*xylE* transcriptional fusion constructs pSbiT_xylE_ and pSbiA_xylE_ were prepared and introduced into S. maltophilia KJ cells to yield KJ(pSbiT_xylE_) and KJ(pSbiA_xylE_). Meanwhile, KJ(pSmeU1_xylE_) was also prepared as a negative control because the *smeU1VWU2X* operon is unexpressed in logarithmically grown S. maltophilia KJ cells (28). The *xylE* gene encodes a catechol-2,3-dioxygenase (C23O); thus, the C23O activities expressed by these constructs were determined. KJ(pSbiT_xylE_) displayed higher C23O activity than KJ(pSmeU1_xylE_), whereas the C23O activity of KJ(pSbiA_xylE_) was comparable to that of KJ(SmeU1_xylE_) (Fig. 3B). The results indicated that the *sbiTRS* operon, but not the *sbiAB* operon, is expressed in logarithmically grown KJ cells.

### SbiT modulates the expression of *sbiAB* operon in a SbiSR-dependent manner.

Given the genomic organization of the *sbiBA*-*sbiTRS* cluster, the role of SbiT in the expression of *sbiSR* and *sbiAB* operons is worth investigating. An in-frame deletion mutant of *sbiT*, KJΔSbiT, was constructed and the transcripts of *sbiB* and *sbiR* in wild-type KJ and KJΔSbiT were comparatively determined by qRT-PCR. Compared to those in wild-type KJ, the *sbiR* transcript levels in KJΔSbiT were slightly higher, but the difference was not statistically significant; however, *sbiB* transcript levels were higher in KJΔSbiT (Fig. 3A). Given the presence of the *sbiTRS* operon, we investigated the involvement of SbiSR TCS in Δ*sbiT*-mediated *sbiB* upregulation. For this purpose, an in-frame deletion mutant of the *sbiTRS* operon, namely, KJΔSbiTRS, was prepared. Compared to KJΔSbiT, the *sbiB* transcript levels in KJΔSbiTRS were significantly lower (Fig. 3A). Collectively, these results suggest that SbiT acts as a negative modulator of the expression of the *sbiAB* operon in a SbiSR-dependent manner.

### Intramembrane protein-protein interaction between SbiT and SbiS.

**FIG 4:**
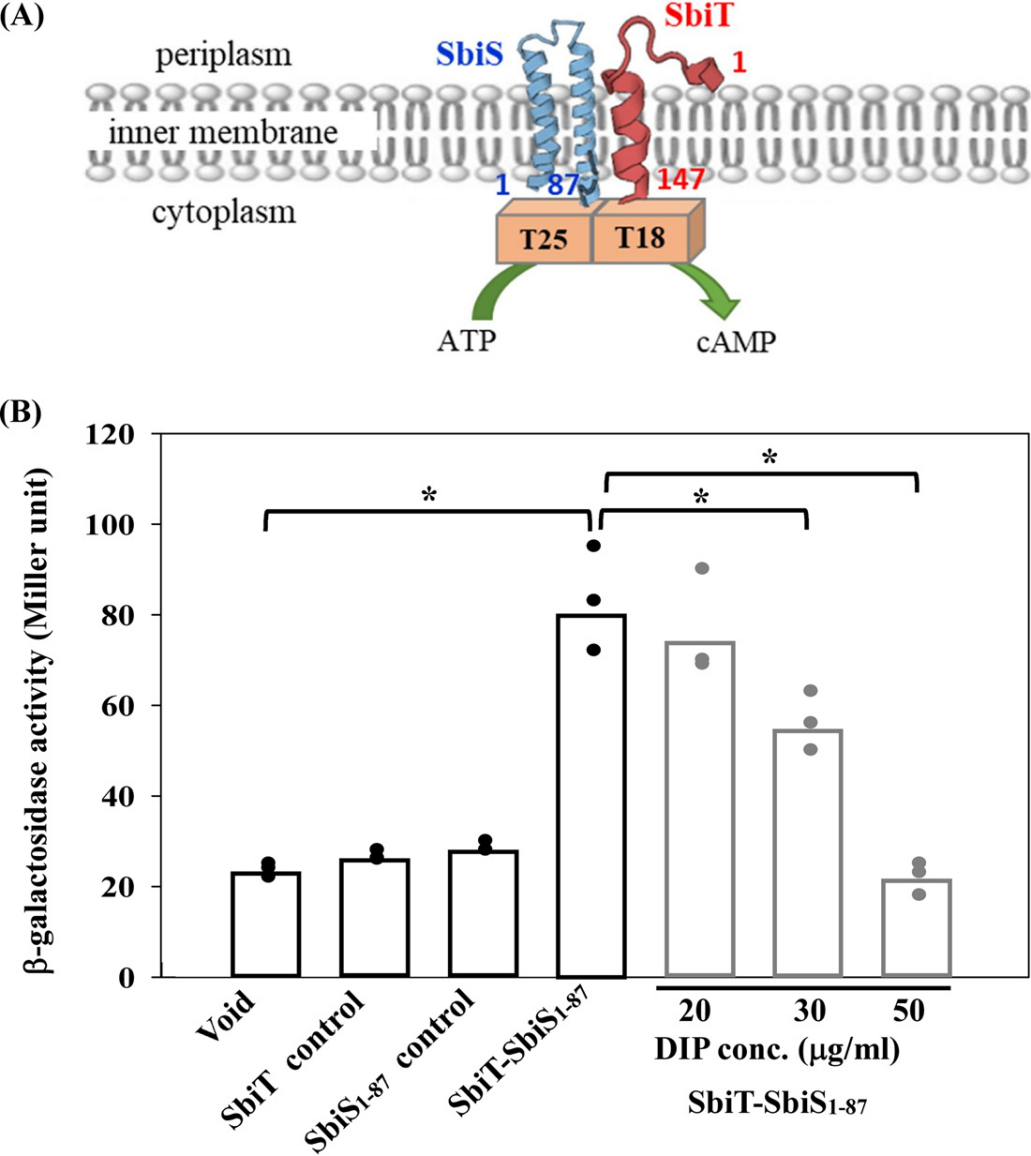
Bacterial adenylate cyclase‐based two‐hybrid analysis of interactions between SbiS and SbiT. (A) Schematic representation of the interaction between T18-SbiT and T25-SbiS_1−87_. Full-length SbiS (1 to 147 aa) and truncated SbiS (1 to 87 aa) were fused in frame to T18 and T25 fragments, respectively. (B) β-galactosidase activity of E. coli DHM1 strain harboring both pUT18- and pKT25-derived plasmids. The plasmids as indicated were cotransformed into E. coli DHM1, and the transformants were grown in LB broth with ampicillin, kanamycin, and isopropyl-β-d-thiogalactopyranoside (IPTG) for 16 h. Beta-galactosidase activity was determined and expressed as Miller units. Each bar represents the mean of values obtained from three independent experiments. Error bars represent the standard error of the mean. *, *P *< 0.01; significance calculated by Student's *t* test. Black bars, void, pUT18 and pKT25; SbiT control, pUT18-SbiT and pKT25; SbiS_1−87_ control, pUT18 and pKT25-SbiS_1−87_; SbiT-SbiS_1−87_, pUT18-SbiT and pKT25-SbiS_1−87_. Gray bars, SbiT-SbiS_1−87_, pUT18-SbiT and pKT25-SbiS_1−87_; DIP concentrations as indicated.

Based on the findings shown in Fig. 3, we hypothesized that SbiT negatively modulates the regulatory circuit of the SbiSR TCS, and the regulatory mechanism does not seem to be at the transcriptional level. SbiT spans the inner membrane with a single transmembrane domain, as predicted by TMHMM server v. 2.0 (https://services.healthtech.dtu.dk/service.php?TMHMM-2.0). Given the localization of SbiT, we hypothesized that SbiT exerts its effects through SbiS via protein-protein interactions. Thus, the intramembrane SbiT-SbiS interaction was investigated using a bacterial two-hybrid system. The *sbiT* gene without the stop codon was cloned into plasmid pUT18, yielding pUT18-SbiT. SbiS is an integral membrane protein with two transmembrane segments (13 to 35 amino acids [aa] and 55 to 77 aa) as predicted by the TMHMM server; thus, the 1 to 261-nucleotide (nt) region of *sbiS* gene was cloned into the plasmid pKT25, generating pKT25-SbiS_1–87_. If the intramembrane regions of SbiS and SbiT interact, the T18 and T25 fragments of Bordetella pertussis CyaA (29) can restore adenylate cyclase activity, leading to increased β-lactamase activity in E. coli DHM1 (Fig. 4A). E. coli DHM1 harboring both plasmids pUT18-SbiT and pKT25-SbiS_1–87_ displayed high levels of β-galactosidase activity compared with the empty vector controls (Fig. 4B), supporting the hypothesis that intramembrane protein-protein interaction occurs between SbiT and SbiS.

### The intramembrane interaction between SbiT and SbiS is affected by intracellular iron levels.

From the above results, we inferred that (i) the *sbiTRS* operon is a member of the Fur regulon, (ii) the Δ*sbiT*-mediated *sbiAB* upregulation is SbiSR TCS dependent, and (iii) an intramembrane protein-protein interaction exists between SbiT and SbiS. Thus, we propose that the SbiT-SbiS interaction functions as a brake to block SbiSR TCS activation. The stimuli that disrupt the SbiT-SbiS interaction can activate SbiSR TCS. We considered the intracellular iron level as the stimulus. Therefore, we assessed the interaction between SbiT and SbiS in the presence of DIP. Compared to the DIP-free counterpart, the β-galactosidase activity was hardly altered in the presence of 20 μg/mL DIP, partially decreased in the presence of 30 μg/mL DIP, and significantly decreased to the level of control when the DIP concentration was increased to 50 μg/mL (Fig. 4B). Collectively, these results indicate that an intramembrane interaction exists between SbiT and SbiS, and this interaction is ferrous iron concentration dependent.

### SbiSR TCS contributes to the stenobactin-mediated ferric iron acquisition.

**FIG 5:**
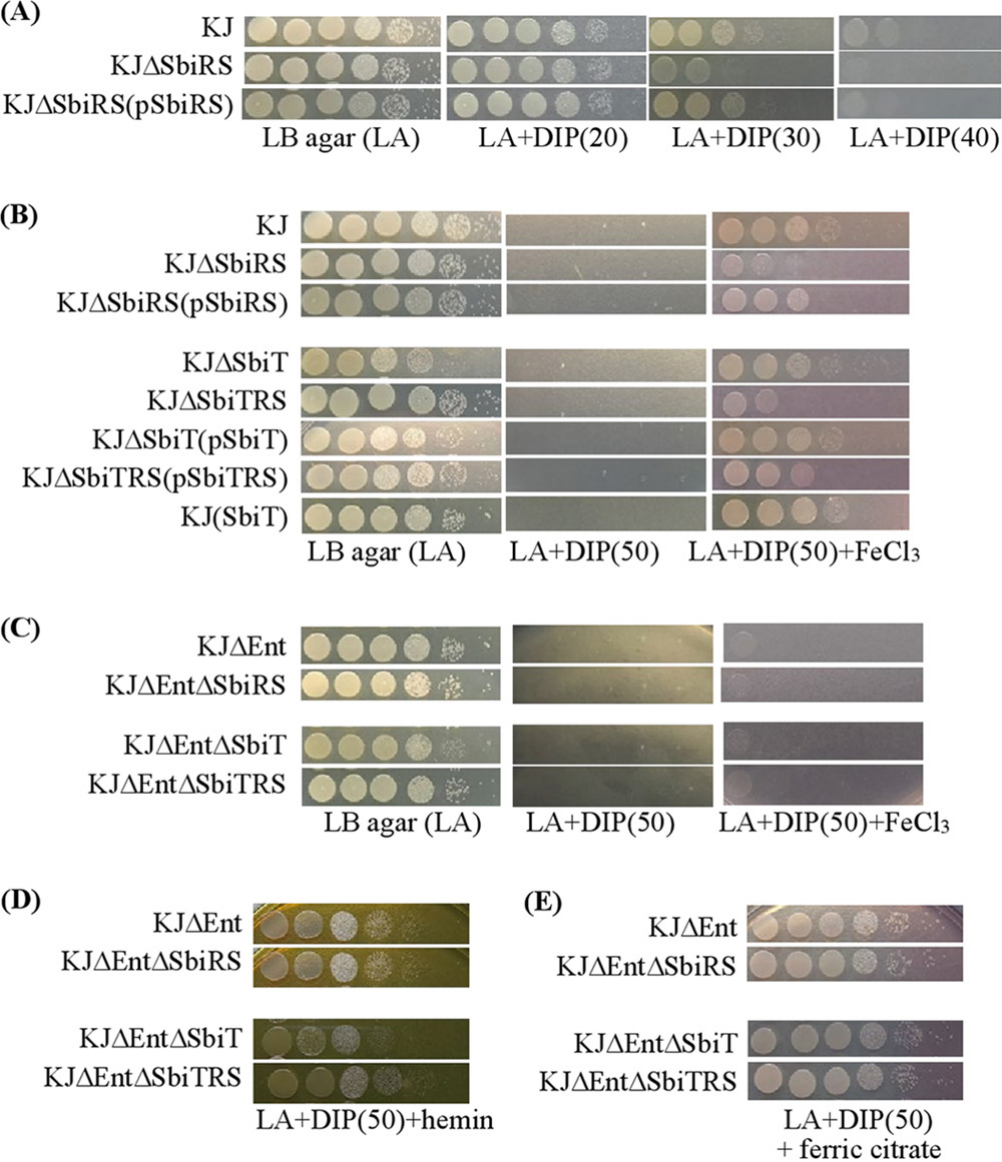
Contribution of SbiSR TCS to the utilization of different iron sources in iron-depleted condition. The logarithmic-phase bacterial cells (2 × 10^5^ CFU/μL) were 10-fold serially diluted. Five microliters of bacterial suspension were spotted onto LB agar plates with different additives as indicated. After a 24-h incubation at 37°C, the viabilities were imaged. The concentrations of additives are as follows: FeCl_3_, 35 μM; hemin, 150 μM; ferric citrate, 110 μM. The numbers in the parentheses indicate the DIP concentration (μg/mL). (A) Role of SbiSR in iron depletion tolerance. (B) Role of SbiSR in ferric iron utilization. (C) Role of stenobactin in SbiSR-mediated ferric iron utilization. (D) Role of SbiSR in hemin utilization. (E) Role of SbiSR in ferric citrate utilization.

Since the *sbiTRS* operon is a member of the Fur regulon and SbiT-SbiS interaction depends on the intracellular iron levels, we presumed that SbiSR TCS promotes iron source acquisition under iron-depleted conditions. Firstly, the impact of *sbiSR* deletion on iron depletion tolerance was assessed. Compared to wild-type KJ, KJΔSbiRS was more sensitive to DIP in a dose-dependent manner (Fig. 5A), supporting our hypothesis. Next, an iron source utilization assay was used to assess the contribution of the SbiSR TCS to the utilization of ferric iron, hemin, and ferric citrate. The paired strains KJ/KJΔSbiRS and KJΔSbiT/KJΔSbiTRS were used for the investigation.

In the KJ/KJΔSbiRS pair, KJΔSbiRS showed compromised growth in DIP- and FeCl_3_-supplemented media, whereas KJ and KJΔSbiRS did not show any observable growth differences on LB agar. Complementation of KJΔSbiRS with *sbiR-sbiS* genes restored the viability to the wild-type level (Fig. 5B), indicating that SbiSR TCS is involved in ferric iron utilization under iron-depleted conditions. For the KJΔSbiT/KJΔSbiTRS pair, we noticed that KJΔSbiT displayed slightly poorer growth than wild-type KJ in an iron-replete LB agar; however, both strains had comparable viability in DIP- and FeCl_3_-supplemented media (Fig. 5B). Like in the case of the KJ/KJΔSbiRS pair, KJΔSbiTRS showed compromised growth in DIP- and FeCl_3_-supplemented media compared to that of KJΔSbiT (Fig. 5B). Given that siderophores are the major tools of ferric iron acquisition in most bacteria (5), we tested the role of stenobactin in SbiST-mediated ferric iron acquisition. For this purpose, *sbiSR*-associated mutants were constructed in KJΔEnt, a stenobactin-null mutant (23), yielding KJΔEntΔSbiT, KJΔEntΔSbiRS, and KJΔEntΔSbiTRS. None of the strains tested showed observable growth in DIP- and FeCl_3_-supplemented media (Fig. 5C). Collectively, the results indicate that SbiSR TCS contributed to stenobactin-mediated ferric iron acquisition under iron-depleted conditions.

Since stenobactin-mediated bacterial growth may shield hemin- and ferric citrate-mediated bacterial growth under iron-depleted conditions, stenobactin-null mutants were used for the assessment of hemin and ferric citrate utilization. Under iron-depleted conditions, KJΔEnt and KJΔEntΔSbiRS showed comparable viability in hemin- and ferric citrate-supplemented media. Similar results were observed for KJΔEntΔSbiT and KJΔEntΔSbiTRS (Fig. 5D and 5E). These observations tentatively ruled out the involvement of SbiSR TCS in the utilization of hemin and ferric citrate.

### SbiSR TCS contributes to siderophore secretion by upregulating the expression of *smeDEF* and *sbiAB* operons.

**FIG 6:**
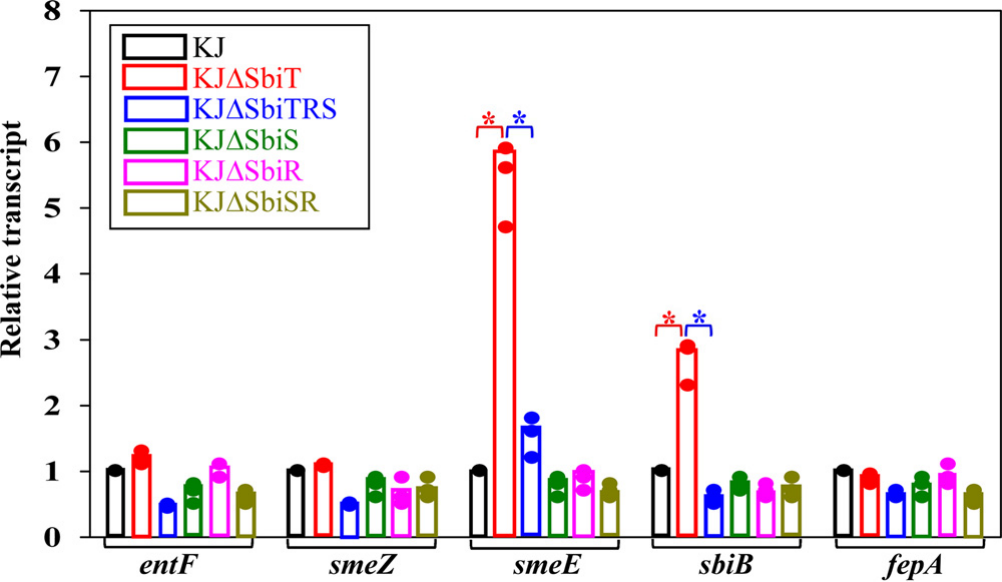
The expression of stenobactin-mediated ferric iron acquisition-related genes in KJ, KJΔSbiT, KJΔSbiTRS, KJΔSbiS, KJΔSbiR, and KJΔSbiSR strains. Overnight culture of the S. maltophilia strains was inoculated into fresh LB at an initial OD_450_ of 0.15. After a 15-h culture, the transcripts as indicated were quantified by qRT-PCR. The relative transcript levels were calculated with the transcript level of KJ cells set as 1. Black dots represent the results of three independent experiments. Bars represent the mean of the values from three independent experiments. *, *P *< 0.01; significance calculated by Student's *t* test.

The above results demonstrate that the SbiSR TCS contributes to stenobactin-mediated ferric iron utilization (Fig. 5A and 5B). Some of the genes involved in stenobactin-mediated ferric iron acquisition in S. maltophilia have been characterized, including stenobactin synthesis genes (*entCEBB’FA* operon) (20), stenobactin secretion genes (*smeYZ*, *smeDEF*, and *SbiAB* operons) (21), and ferri-stenobactin uptake TonB-dependent outer membrane receptor FepA (22). To assess whether these genes are members of the SbiSR TCS, we compared their expression levels in KJ, KJΔSbiT, KJΔSbiTRS, KJΔSbiR, KJΔSbiS, and KJΔSbiSR using qRT-PCR. Among the genes tested, *smeE* and *sbiB* transcript levels were upregulated in KJΔSbiT and were almost equal to the wild-type levels in KJΔSbiTRS (Fig. 6), indicating that *smeDEF* and *sbiAB* operons are members of the SbiSR regulon. Meanwhile, we also noticed that the transcript level of all of the genes tested were not significantly altered in KJΔSbiS, KJΔSbiR, and KJΔSbiSR (Fig. 6), supporting the conclusion that the activation of the SbiSR regulon results from *sbiT* deletion.

## DISCUSSION

The primary objective of this study was to identify a novel regulatory system, which is the member of the Fur regulon and is involved in iron homeostasis in S. maltophilia. Using transcriptome analysis and experimental approaches, we identified a novel TCS, SbiS/SbiR, which contributes to stenobactin-mediated ferric iron acquisition under iron-depleted condition. *SbiR*, *sbiS*, and the upstream gene, *sbiT*, together form an operon. SbiT, a 147-aa inner membrane protein, downregulates the SbiS/SbiR pathway. The involvement of small proteins (<200 aa) in TCS modulation has been reported in several microbes (30). PmrD, a connector, connects PhoPQ and PmrAB TCSs in Salmonella enterica (31). PmrD, a member of PhoPQ regulon, can bind to phosphorylated PmrA (an RR), preventing its dephosphorylation by its cognate SK PmrB (32). CpxP interacts with CpxA (an SK) to inhibit the CpxA/CpxR pathway (33). B1500 forms a complex with PhoQ (an SK) to activate the PhoPQ TCS (34). These small proteins can be located in the cytoplasm (such as PmrD), periplasm (such as CpxP), or the inner membrane (such as B1500). Small proteins generally regulate TCS through protein-protein interactions to adapt to rapid environmental changes (35). In this study, we provide the first example of a small protein, SbiT, that acts as a TCS modulator in S. maltophilia. SbiT, like B1500, is an inner membrane protein that forms a complex with the sensor kinase. Nevertheless, the outcome of the SbiT-SbiS interaction is different from that of the B1500-PhoQ interaction: a negative impact on TCS activation by the former and a positive impact by the latter. Whether the SbiT-mediated inhibition results from the inhibition of SbiS sensing iron levels or from the prevention of SbiS autophosphorylation, phosphatase, or phosphotransferase activity remains to be investigated.

An interesting result that we noted is that the *sbiT* mutant (KJΔSbiT) displayed growth impair on LB agar but not on LB agar supplemented with DIP and FeCl_3_ (Fig. 5B). Deletion of *sbiT* may result in SbiSR TCS activation due to the loss of SbiT-SbiS interaction, even in an iron-replete condition. Given that the *sbiTRS* operon is a member of Fur regulon and activated in response to iron depletion, we can reason that SbiSR TCS activation may favor intracellular iron level increase and KJΔSbiT appears to be in an iron-overload situation. This may be the reason why *sbiT* mutant exhibits a growth compromise on LB agar.

The ability of the SK to specifically respond to changes in iron levels has been described in some microorganisms, such as in case of BqsRS in P. aeruginosa (36), PmrAB in Salmonella enterica serovar *Typhimurium* (37), and FirRS in Haemophilus influenzae (38). In these instances, SK generally senses iron levels via certain amino acid residues (such as RExxE, HExxE, and DYRED motifs), triggering TCS activation. However, we did not find any iron-sensing motifs in SbiS. Thus, we wondered whether iron binds to SbiT to raise the SbiT-SbiS interaction. To test this notion, we evaluated the impact of SbiT overexpression from a plasmid on the SbiSR activity. If SbiT bound with iron is a critical factor for SbiT-SbiS interaction, the surplus SbiT from plasmid pSbiT may sequester iron and then break down SbiT-SbiS interaction, which may lead to SbiSR partial activation, even in an iron-replete condition. Thus, we can expect that the viability of KJ(pSbiT) in LB agar may be compromised, phenotypically mimicking KJΔSbiT. However, the results did not support the hypothesis since KJ(pSbiT) displayed comparable viability with wild-type KJ in LB agar (Fig. 5B). Collectively, we can conclude that the SbiT-SbiS interaction is modulated by the availability of intracellular iron, but the underlying mechanism by which iron influences this interaction remains unclear.

**FIG 7:**
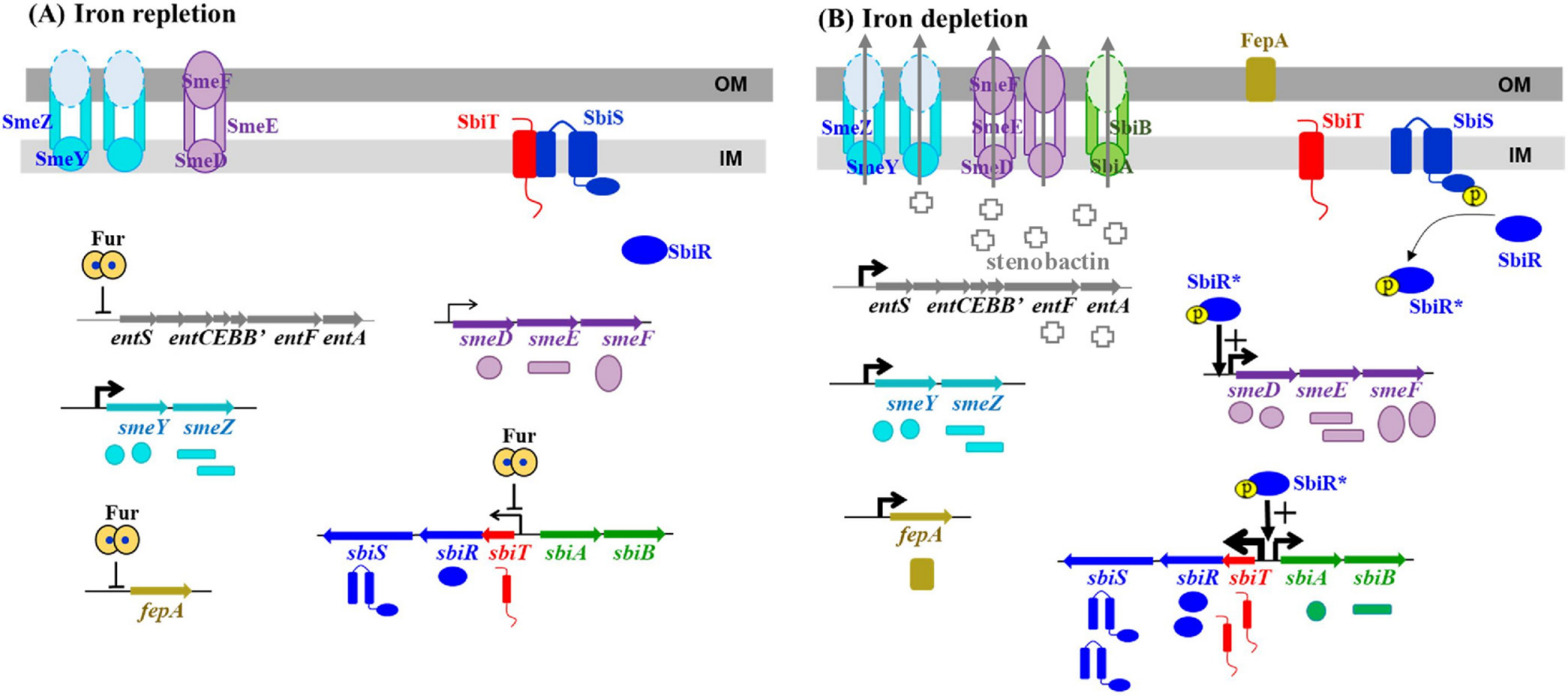
Model of Fur-SbiT-SbiSR-SmeDEF/SbiAB regulatory circuit in S. maltophilia. (A) In iron-replete condition, Fur-ferrous iron complex partially represses *sbiTRS* operon expression. The expressed SbiT and SbiS show intramembrane protein-protein interaction, which blocks SbiSR TCS activation. (B) In iron-depleted condition, the Fur regulon is derepressed and SbiT-SbiS interaction disappears. Ceasing of repression by Fur regulon leads to stenobactin synthesis and upregulation of the *sbiTRS* operon. Meanwhile, the loss of the SbiT-SbiS interaction results in SbiSR TCS activation. Phosphorylated SbiR upregulates the expression of *sbiAB* and *smeDEF* operons. SmeDEF and SbiAB pumps are the known stenobactin secretion outlets.

Siderophore-mediated ferric iron acquisition is regarded as the most powerful and efficient ferric iron acquisition system in bacteria. In response to iron depletion stress, siderophore synthesis, siderophore secretion, ferric iron capture, ferri-siderophore uptake, and ferric iron detachment are regulated in a coordinated manner to ensure successful siderophore-mediated ferric iron utilization (4). In most Gram-negative bacteria, Fur is the global transcriptional regulator used to cope with stress through the intricate regulatory network. It is therefore reasonable to consider that many regulatory circuits are under the regulation of Fur. Based on the results of this study, we propose a novel Fur-SbiT-SbiSR-SmeDEF/SbiAB regulatory circuit. In iron-replete conditions, Fur partially represses the *sbiTRS* operon. The *sbiTRS* operon displayed moderate expression in logarithmically grown cells. The intramembrane protein-protein interaction between SbiT and SbiS maintains the SbiSR TCS in a resting state (Fig. 7A). Therefore, SbiSR TCS is inactive under iron-replete conditions via the regulation of Fur and SbiT. Fur represses *sbiSR* expression at the transcriptional level, and SbiT represses SbiS activation at the posttranslational level through protein-protein interaction. In iron-depleted conditions, the *sbiTRS* operon is no longer repressed by Fur and the SbiT-SbiS interaction disappears, resulting in the activation of SbiSR TCS. Activated SbiR upregulates the expression of the *smeDEF* and *sbiAB* operon (Fig. 7B). SmeDEF and SbiAB are efflux pumps used for stenobactin secretion (21). Although the genes involved in stenobactin synthesis and ferri-stenobactin uptake seem not to be the members of SbiSR TCS, we can expect them to be upregulated in a coordinated manner in response to iron depletion via other unidentified Fur-involved regulatory circuits, since these genes are the members of Fur regulon (Table 1; Fig. 7B). These regulatory circuits finely modulate stenobactin-mediated ferric iron utilization and ensure iron acquisition under iron-depleted conditions.

## MATERIALS AND METHODS

### Bacterial strains, media, plasmids, and primers.

The bacterial strains and plasmids used are listed in Table S2 in the supplemental material. The primers used were listed in Table S3 in the supplemental material.

### Transcriptome analysis.

In our previous studies, we found that the stenobactin synthesis gene *entF* is upregulated in logarithmic-phase KJΔFur cells. However, when we tried to determine the secreted stenobactin from the cell-free culture supernatants using the CAS activity assay, interestingly, obvious CAS activity was detected from stationary-phase KJΔFur cells but not from the logarithmic-phase KJΔFur cells (20). This experience made us design the transcriptome analysis for the stationary-phase cells, intending to observe a more detailed iron limitation response.

Overnight cultures of KJ and KJΔFur were subcultured into fresh LB broth with an initial optical density at 450 nm (OD_450_) of 0.15 and further incubated at 37°C for 15 h. Total RNA isolation, rRNA depletion, adapter-ligated cDNA library construction and enrichment, and cDNA sequencing were performed as described previously (39). The output R1 reads were mapped to the genome of K279a (40) using bwa v. 0.7.1. The gene mapping reads were counted using htseq v. 0.13.5 with arguments stranded reverse and mode union. The total number of reads per gene between both strains was normalized by transcripts per kilobase million (TPM) values. The RNA-seq data have been deposited in GenBank under BioProject accession number PRJNA876818.

### Reverse transcription-PCR and operon verification.

DNA-free RNA was purified from KJΔFur cells and reverse-transcribed into cDNA using the SbiR-C primer set targeting the *sbiR* gene. cDNA was used as the specimen for PCR using the primer sets SbiTQ102-F/R and SbiRQ94-F/R (see Table S3). The SbiRQ94-F/R primer sets were used as negative controls to check for DNA contamination. The PCR products were separated by electrophoresis on a 2% agarose gel and visualized by staining with ethidium bromide.

### Quantitative real-time PCR.

DNA-free RNA was prepared from 15-h cultured bacterial cells and converted to cDNA using a high-capacity cDNA reverse transcription kit (Applied Biosystems). cDNA (1:100 dilution), primers, and TaqMan Universal PCR master mix (Applied Biosystems) were used. The primers used are listed in Table S3. qRT-PCR was performed using the ABI Prism 7000 sequence detection system (Applied Biosystems) according to the manufacturer’s instructions. All gene expression levels were normalized to those of 16S rRNA as the internal control. Fold change was calculated using the ΔΔ*C_T_* method (41). All experiments were performed in triplicate.

### Construction of promoter-*xylE* transcriptional fusions.

The DNA segment containing the promoter regions of *sbiT* and *sbiA* was amplified by PCR using the primer set SbiTN-F/R and cloned into plasmid pRKXylE (42) in two different orientations, yielding pSbiT_xylE_ and pSbiA_xylE_, respectively.

### C23O activity determination.

The activity of the catechol-2,3-dioxygenase encoded by the *xylE* gene was measured as previously described (42). One unit of enzyme activity (U) was defined as the amount of enzyme that converts 1 nmol of catechol per minute. The specific activity (U/OD_450_) of the enzyme was defined as units per OD_450_ unit of cells. All experiments were performed in triplicate.

### Mutant construction.

The allelic replacement strategy was used to construct in-frame deletion mutants as previously described (43). Briefly, two DNA fragments containing ~500 bp of the N terminus and C terminus of the intended deletion region were amplified by PCR using the chromosome of S. maltophilia KJ as a template. Table S3 lists the primers used. The two PCR amplicons were subsequently cloned into pEX18Tc to generate recombinant plasmids pΔSbiT, pΔSbiRS, pΔSbiTRS, pΔSbiS, pΔSbiR, and pΔSbiSR (Table S2). The pEX18Tc-derived recombinant plasmids were then introduced into S. maltophilia KJ via conjugation. Transconjugant selection and double crossover mutant confirmation were performed as described previously (43).

### Construction of complementation plasmids.

The complementation plasmids, pSbiT, pSbiRS, and pSbiTRS, were constructed by amplifying *sbiT*, *sbiRS*, and *sbiTRS* from S. maltophilia KJ using primers SbiT-F/R, SbiRS-F/R, and SbiTRS-F/-R, respectively, and cloning them into pRK415 under the control of the *lacZ* promoter. The primers used and the resultant plasmids for complementation are summarized in Tables S3 and S2, respectively.

### Bacterial adenylate cyclase two-hybrid assay.

The 1 to 441 nt of the *sbiT* gene (corresponding to the 1 to 147 aa of SbiT protein) and 1 to 261 nt of the *sbiS* gene (corresponding to the 1 to 87 aa of SbiS protein) were amplified by PCR using the primer sets SbiT18-F/SbiT18-R and SbiS25_1-87_-F/SbiS25_1-87_-R, respectively (see Table S3). The 441-bp and 261-bp PCR amplicons were cloned into vectors pUT18C and pKT25, respectively, generating recombinant plasmids pUT18-SbiT and pKT25-SbiS_1–87_ (see Table S2). Plasmids pUT18-SbiT and pKT25-SbiS_1–87_ were then cotransformed into the Δ*cya* strain (E. coli DHM1). β-galactosidase assays were performed as previously described (24). The experiments were performed in triplicate.

### Iron source utilization assay.

An iron source utilization assay was used to assess the ability of the bacterial cells to utilize exogenous iron to support growth under iron-depleted condition. In our previous studies, we had observed that KJ cells were unable to grow in a medium containing 50 μg/mL 2,2′-dipyridyl (DIP) unless an exogenous iron source was provided (20). The logarithmic-phase bacterial cells tested were adjusted to a concentration of 2 × 10^5^ CFU/μL, followed by 10-fold serial dilutions. A 5-μL volume of each dilution was spotted onto the plates as indicated. Bacterial cell growth was observed after a 24-h incubation at 37°C.

### Data availability.

The RNA-seq data have been deposited in GenBank under BioProject accession number PRJNA876818.
