# Supplementary material for: The sbiTRS Operon Contributes to Stenobactin-Mediated Iron Utilization in Stenotrophomonas maltophilia
Source: Microbiol Spectr. 2022 Dec 1;10(6):e02673-22. doi: 10.1128/spectrum.02673-22 (PMC9769818; doi:10.1128/spectrum.02673-22)
Supplement: Supplemental file 2 — Table S2, Table S3. Download spectrum.02673-22-s0002.pdf, PDF file, 0.6 MB [file spectrum.02673-22-s0002.pdf]

Table S2 Bacterial strains and plasmids used in this study

Strain, plasmid, or primer	Genotype or properties	Reference
S. maltophilia		
KJ	A clinical *S. maltophilia* isolate	1
KJΔSbiT	*S. maltophilia* KJ mutant of *sbiT* gene; *ΔsbiT*	This study
KJΔSbiR	*S. maltophilia* KJ mutant of *sbiR* genes; *ΔsbiR*	This study
KJΔSbiS	*S. maltophilia* KJ mutant of *sbiS* genes; *ΔsbiS*	This study
KJΔSbiRS	*S. maltophilia* KJ mutant of *sbiRS* genes; *ΔsbiRS*	This study
KJΔSbiTRS	*S. maltophilia* KJ mutant of *sbiTRS* genes; *ΔsbiTRS*	This study
KJΔFur	*S. maltophilia* KJ mutant of *fur* gene; *Δfur*	2
E. coli		
DH5α	F- φ80d/*acZΔM15* Δ(*lacZYA-argF*)*U169 deoR recA1 endA1 hsdR17* (r_k^- m_k^+) *phoA supE44λ thi-1 gyrA96 relA1*	Invitrogen
S17-1	λ*pir*$^+$ mating strain	3
**Plasmids**		
pEX18Tc	*sacB oriT*, Tc^r	4
pRK415	Mobilizable broad-host-range plasmid cloning vector, RK2 origin; Tc^r	5
pΔSbiT	pEX18Tc with an internal-deletion *sbiT* gene; Tc^r	This study
pΔSbiR	pEX18Tc with an internal-deletion *sbiR* gene; Tc^r	This study
pΔSbiS	pEX18Tc with an internal-deletion *sbiS* gene; Tc^r	This study
pΔSbiRS	pEX18Tc with partial N-terminus of *sbiR* gene and partial C-terminus of *sbiS* gene; Tc^r	This study
pΔSbiTRS	pEX18Tc with an internal-deletion *sbiR and sbiS* genes; Tc^r	This study
pSbiT	pRK415 with an intact *sbiT* gene	This study
pSbiRS	pRK415 with intact *sbiRS* genes	This study
pSbiTRS	pRK415 with intact *sbiTRS* genes	This study
$pSbiT_{xylE}$	pRK415 with a 319-bp DNA fragment upstream from the *sbiT* start codon and a P_{sbiT}::*xylE* transcriptional fusion	This study
$pSbiA_{xylE}$	pRK415 with a 312-bp DNA fragment upstream from the *sbiA* start codon and a P_{sbiA}::*xylE* transcriptional fusion	This study
$pSmeU1_{xylE}$	pRK415 with a 425-bp DNA fragment upstream from the	6

	smeU1 start codon and a P_{smeU1}::*xylE* transcriptional fusion	
pUT18-SbiT	pUT18 with a translational-fusion SbiT-T18	This study
pKT25-SbiS$_{1-87}$	pKT25 with a translational-fusion T25-SbiS$_{1-87}$	This study

1. Hu RM, Huang KJ, Wu LT, Hsiao YJ, Yang TC. 2008. Induction of L1 and L2 beta-lactamases of *Stenotrophomonas maltophilia*. Antimicrob Agents Chemother 52:1198-1200.
2. Liao CH, Chen WC, Li LH, Lin YT, Pan SY, Yang TC. 2020. AmpR of *Stenotrophomonas maltophilia* is involved in stenobactin synthesis and enhanced β-lactam resistance in an iron-depleted condition. J Antimicrob Chemother 75:3544-3551.
3. Simon R, O'Connell M, Labes M, Puhler A. 1986. Plasmid vector for the genetic analysis and manipulation of *Rhizobia* and other Gram-negative bacteria. Methods Enzymol 118:640-659.
4. Hoang TT, Karkhoff-Schweizer RR, Kutchma AJ, Schweizer HP. 1998. A broad-host-range Flp-FRT recombination system for site-specific excision of chromosomally-located DNA sequences: application for isolation of unmarked *Pseudomonas aeruginosa* mutants. Gene 212:77-86.
5. Keen NT, Tamaki S, Kobayashi D, Trollinger D. 1998. Improved broad-host-range plasmids for DNA cloning in gram-negative bacteria. Gene 70:191-197.
6. Chen CH, Huang CC, Chung TC, Hu RM, Huang YW, Yang TC. 2011. Contribution of resistance-nodulation-division efflux pump operon *smeU1-V-W-U2-X* to multidrug resistance of *Stenotrophomonas maltophilia*. Antimicrob Agents Chemother 55:5826-5833.

Table S3 PCR primers used in this study

Primer	Sequence (5'→3')	Purpose
SbiR-C	CCGAGAATGTCAGCGATTTC	Operon check
SbiTQ102-F	ATCACGGTGGAGCAAGCT	Operon check
SbiTQ102-R	CACCAGCGTGTGCTGAAG	qRT-PCR
SbiBQ105-F:	GCAGGTCGAGTCACAGATCA	qRT-PCR
SbiBQ105-R:	ACGACCAGCGTTTCAATCTC	
EntFQ109-F:	CGTGATGTGTCCGTCGAG	qRT-PCR
EntFQ109-R:	GCACCTGTTGGCTGATCC	
SmeZQ93-F	GATCCAGAAGGTGGTGCAGA	qRT-PCR
SmeZQ93-R	ATCAGCGTGTAGCGCCAGT	
SmeEQ108-F	TCAAGCCGCTGAAGAAGG	qRT-PCR
SmeEQ108-R	GCTGGTAGCTTTCGCTGGT	
FepAQ97-F	AACCGCATGTACCGCAAC	qRT-PCR
FepAQ97-R	AGTTGTTGACCGCCTCCA	
16S rDNA-F	GACCTTGCGCGATTGAATG	qRT-PCR
16S rDNA-R	CGGATCGTCGCCTTGGT	
SbiRQ94-F	AACACACCGGTGATCATGC	Operon check
SbiRQ94-R	AGGGCTTGGCCACATAGTC	qRT-PCR
SbiTN-F	CAAAGCTTGTGACCGCAGAA	pΔSbiT construction
SbiTN-R	GATCTAGACCAGTGACGCCA	
SbiTC-F	GCTCTAGAAGAAGCACAGA	
SbiTC-R	GCGGTACCAGCTCACTCAGGA	
SbiRN-F	GCTCTAGAAGAAGCACAGA	pΔSbiRS construction
SbiRN-R	GCGGTACCAGCTCACTCAGGA	
SbiSC-F	CGGAGCTCGCGCGTTCGAGA	
SbiSC-R	GCGAATTCGATGGCATGGCCGA	
SbiRN-F	GCTCTAGAAGAAGCACAGA	pΔSbiR construction
SbiRN-R	GCGGTACCAGCTCACTCAGGA	
SbiRC-F	GGGGTACCACGTCAGCAA	
SbiRC-R	AGGAGCTCAACGTGGCCA	
SbiRC-F	GGGGTACCACGTCAGCAA	pΔSbiS construction
SbiRC-R	AGGAGCTCAACGTGGCCA	
SbiSC-F	CGGAGCTCGCGCGTTCGAGA	
SbiSC-R	GCGAATTCGATGGCATGGCCGA	
SbiTN-F	CAAAGCTTGTGACCGCAGAA	pΔSbiTRS construction
SbiTN-R	GATCTAGACCAGTGACGCCA	

SbiSC-F	CGGAGCTCGCGCGTTCGAGA	
SbiSC-R	GCGAATTCGATGGCATGGCCGA	
SbiTN-F	CAAAGCTTGTGACCGCAGAA	pSbiT construction
SbiTC-R	GCGGTACCAGCTCACTCAGGA	
SbiRS-F	CCGAAGCTTAAGAAGCACAGACCCTGGAA	pSbiRS construction
SbiRS-R	GAGTCTAGATGGAGATTGCGTGGAGAAA	
SbiTRS-F	ATGAAGCTTGGAGCCACTGTGGAGAT	pSbiTRS construction
SbiTRS-R	GAGTCTAGATGGAGATTGCGTGGAGAAA	
SbiT18-F	CGACCAGCTCTAGAGATGCTTGCCA	protein-protein interaction
SbiT18-R	ATGCCCGTTGAATTCACCCGGTCCT	
SbiS25$_{1\text{-}87}$-F	CTTTCTAGATGAACCGATGAACC	protein-protein interaction
SbiS25$_{1\text{-}87}$-R	ACCGAATTCACGGGGTGATGAT	
